# Prevalence of human parvovirus B19 in Chinese plasma pools for manufacturing plasma derivatives

**DOI:** 10.1186/s12985-015-0396-z

**Published:** 2015-10-06

**Authors:** Junting Jia, Yuyuan Ma, Xiong Zhao, Yi Guo, Chaoji Huangfu, Chi Fang, Rui Fan, Maomin Lv, Huiqiong Yin, Jingang Zhang

**Affiliations:** Laboratory for Viral Safety of National Centre of Biomedical Analysis, Beijing Institute of Transfusion Medicine, No. 27 Taiping road, Haidian District, Beijing, 100850 China; Shaanxi Blood Center, Xi’an, China

**Keywords:** Human parvovirus B19, Quantitative PCR (qPCR), Source plasma pools

## Abstract

**Background:**

Human parvovirus B19 (B19V) is a frequent contaminant of blood and plasma-derived medicinal products. To ensure the quality and safety of plasma-derived products, European regulations, Plasma Protein Therapeutics Association (PPTA) standard and FDA guidelines require testing of manufacturing plasma for parvovirus B19 DNA to limit the load of this virus. In China, however, there have been no related documentation and technical guiding principles for monitoring B19V, moreover, an adequate level of information on the prevalence of B19V in Chinese plasma donations is not available.

**Findings:**

By using an in-house quantitative polymerase chain reaction (qPCR) assay adapted for all three genotypes of B19V, 235 source plasma pools from three regional different Chinese manufacturers of blood products were screened and quantified. Results showed that 71.91 % (169/235) of plasma pools were contaminated by B19V, with the concentrations of 5.18 × 10^2^–1.05 × 10^9^ IU/mL. Approximately 31.95 % of the DNA-positive plasma pools were only moderately contaminated (<10^4^ IU/mL), while 68.05 % contained >10^4^ IU/mL.

**Conclusions:**

The high level of B19V in plasma pools could present a great risk in plasma derivatives. Therefore, the implementation of B19V NAT (Nucleic Acid Testing) assays capable of detecting all B19V genotypes and discard donations with high titer B19V DNA for Chinese blood products manufacturers seems to be necessary.

## Findings

Human parvovirus B19 (B19V), a widespread human pathogen that be associated with a broad range of clinical manifestations, including erythema infectiosum (also called fifth disease), arthritis, transient aplastic crisis (TAC), chronic anemia, hydrops fetalis, and fetal death, can be transmitted via the administration of contaminated blood and plasma-derived products [1–5].

Due to the difficulty in removing and inactivating B19V, the most effective measure for mitigating the risk of B19V transmission through plasma derivatives should be limiting the virus load in the manufacturing plasma pools. Since the early 21st century, U.S. Food and Drug Administration (FDA), European Pharmacopoeia (Ph. Eur.) and the Plasma Protein Therapeutics Association (PPTA) have developed a list of standards, proposing a limit of 10^4^ IU/ml for levels of B19V DNA in source plasma pools destined for manufacturing some or all kinds of plasma-derived products [6–10]. After the introduction of NAT (nucleic acid testing), both the prevalence and level of B19V DNA within plasma pools and their resulting products were significantly lowered [11]. B19V is now formally subdivided into three distinct genotypes (1, 2, 3), which were defined as having approximately 10 % divergence in overall DNA sequence [12]. Since all genotype variants can be contaminants of blood and blood-derived products, so the NAT assay as an in-process test used in the manufacturing of plasma-derived products must be able to detect all genotype variants [13, 14].

In China, there have been no specific documentation and technical guidelines for monitoring B19V. There are about 33 manufacturers for blood products in China, however, B19V NAT screening of plasma has never been implemented in the manufacturing of plasma-derived products. Published data on the prevalence of B19V DNA in plasma pools are limited to the study by Zhang et al., whose investigation was restricted to 142 plasma pools from two of these manufacturers (one was located in the south of China, whereas the other’s location was not mentioned), indicating that the positive rates of B19V DNA in plasma pools were relatively high (54.23 %) [15].

In this study, we aimed to test a total of 235 source plasma pools, collected between 2008 and 2013, from other three Chinese blood products manufacturers (Manufacturer A, B and C, respectively located in central, northern and south-western China). The collections of plasma samples were approved by the National Health and Family Planning Commission of the People’s Republic of China, and all of the donators provided informed consent. Each batch of the plasma pools consisted of 2000 to over 8300 donations. Each donation mixed in the plasma pools was tested for aminotransferase (ALT), anti-syphilis, anti-HIV 1/2, HBsAg and anti-HCV before pooling. All the donations were confirmed to be qualified according to the requirements of Pharmacopeia of the People’s Republic of China [16]. Viral DNA was extracted from a volume of 200 μL pooled source plasma by using the High Pure Viral Nucleic Acid Kit (Roche Diagnostics, Mannheim, Germany) according to manufacturer’s instructions. Nucleic acids were eluted from the filter column with 50 μL of nuclease-free double-distilled water and were stored at −20 °C until further use.

**Fig. 1:**
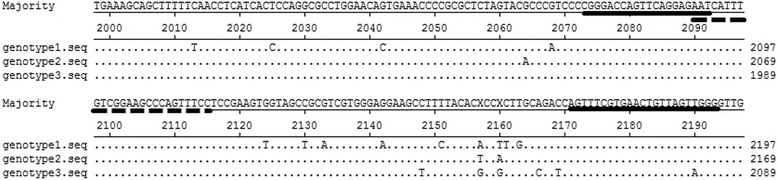
Primers and probe-binding sequence of the in-house B19 DNA assays. The reference sequences of three B19 virus genotypes are aligned. Primers sequences were underlined with *solid lines*, and probe sequence was underlined with *dashed line*. B19 Genotype 1 sequence M13178 is used as reference sequence. For B19 Genotype 2 and 3, the Lali strain [GenBank: AY044266] and V9 strain [GenBank: NC-004295] are included in this alignment

**Fig. 2:**
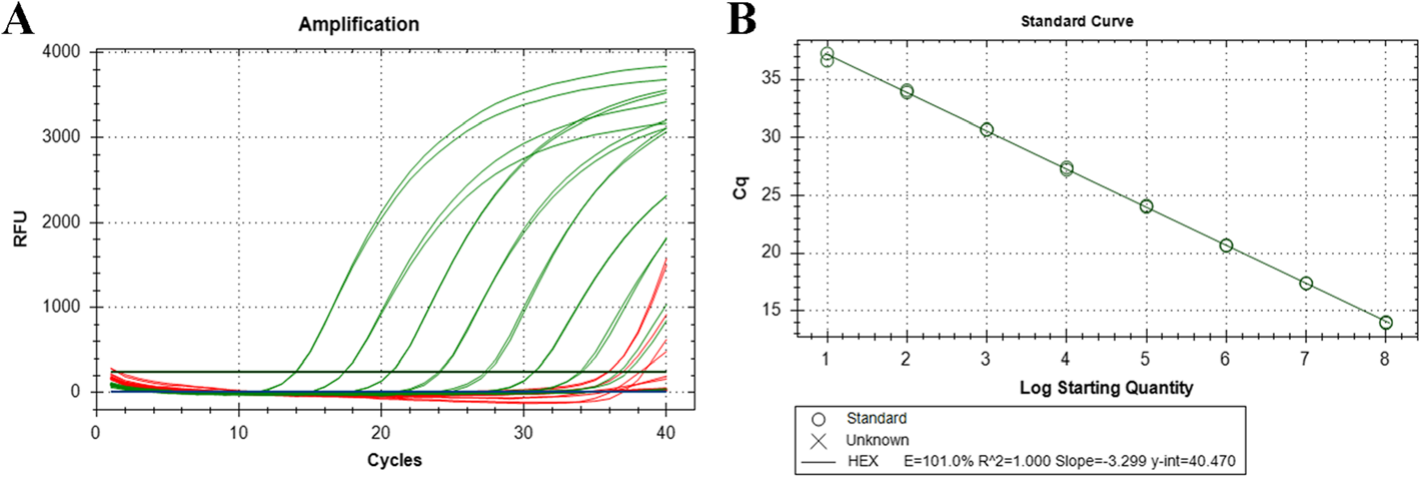
Standard curve of the real-time qPCR assay for B19V DNA. **a** Amplification plots showing the testing in duplicate of a 10-fold dilution series containing standard plasmid of B19V from 1 × 10^8^ to 1 × 10^1^ template copies per μL, and the IC plasmid at 120 copies per reaction. **b** Real-time PCR standard curve generated from plasmid DNA amplification plots(a). The x-axis represents B19V standard plasmid in 10-fold dilutions and the y-axis represents the fluorescence data used for Cq determinations. The assay was linear in the range of 1 × 10^8^ to 1 × 10^1^ template copies per μl, with an R^2^ of 1.000, the slope value of −3.299 and reaction efficiencies of 101.0 %

Quantification of B19 DNA was performed by using an in-house developed qPCR assay adapted for all three genotypes of B19V. Primers B19F (5′-CGGGACCAGTTCAGGAGAAT-3), B19R (5′-CCCAACTAACAGTTCACGAAACT-3′) and probe B19P (5′-AATCATTTGTCGGAAGCCCAGTTTCC-3′) were designed to specifically amplify a 121-bp fragment derived from the NS1 gene of B19V (nucleotides 2073–2193, GenBank accession no. M13178.1) (Fig. 1). An exogenous competitive IC (internal control) contained the identical sequence of the B19 target sequence but with an altered probe hybridization site (ICP: 5′-AATTCGATCTTGGACCAGCAGTTCTC-3′), was included in each sample to prevent false negative results. This allows co-amplification of B19V and IC and co-detection of HEX (5-Hexachlorofluorescein) or FAM (6-carboxyfluorescein) labeled probes respectively. Three microliters of DNA was added to a reaction mixture (in a final volume of 20 μL) containing 10 μL of Platinum Quantitative PCR SuperMix-UDG (Invitrogen Life Technologies, Paisley, United Kingdom), 500nM of each primer, and 250nM of each probe for amplification of DNA from the B19V and IC. Amplification and detection were carried out on the Bio-Rad MiniOpticon Real-Time PCR System (Bio-Rad Laboratories, Hercules, USA) with the following thermal cycle conditions: 2 min of incubation at 50 °C, 2 min of pre-denaturation at 95 °C, 40 cycles in two steps each (95 °C for 9 s, 60 °C for 30 s). Standard curves were generated with serial log 10 dilutions ranging from 10^8^ to 10^1^ copies per μL of the B19V standard plasmid containing the 121-bp PCR product. One hundred and twenty copies of the IC plasmid were included in each well and all samples were analyzed in duplicate. The assay was shown to be linear over this eight orders of magnitude. The IC DNA could be detected when B19V plasmid lower than 10^3^ copies per μL and in the no template control (NTC) well (Fig. 2). The performance of this NAT assay was evaluated with the 1st WHO International Reference Panel for Parvovirus B19 Genotypes for NAT based assays (NIBSC code: 09/110). This assay was able to detect all three B19 virus genotypes with the sensitivity of 5 copies per reaction, and the conversion ratio from copies to IU is 5.08:1, indicating that 5.08 B19V copies/mL measured by our real-time qPCR assay was equivalent to 1 IU/mL.

As indicated in Table 1, 72 % (169/235) of the samples contained detectable levels of B19V and the amount of DNA ranged from 5.18 × 10^2^ to 1.05 × 10^9^ IU/mL plasma. Forty-nine percent (115/235) of plasma pools contained B19V DNA at concentrations exceeding 10^4^ IU/mL. In the case of manufacturer B, the positive rate of B19V DNA in plasma pools was 100 %, higher than that from manufacturer A (73.76 %) and manufacturer C (60.81 %). Positive rates of B19V DNA in plasma pools from three manufacturers were significantly different (*χ*^*2*^ = 12.566, *P* = 0.002 < 0.05, chi-square test). Above results showed that both of the prevalence and levels of B19V DNA in plasma pools tested in our study were higher than that of previous reports in China (54.20 %) and investigations in other countries before NAT was introduced (56.10 % ~ 59.68 %) [15, 17, 18]. Besides, there were large differences among the positive rates of industrial plasma pools of five manufactures (three in our study and the other two in previous report) in China, ranging from 30 to 100 %. These diversities may reflect geographic differences in the spread of the virus in diverse parts of the world, methodological differences in diagnostic procedures, differences in sample size, or differences in number of plasma units within each pool sample.

**Table 1:** Distribution of the B19V DNA load in source plasma pools

B19V DNA load (IU/mL)	Number of lots (%)			
	Manufacturer A	Manufacturer B	Manufacturer C	Total
10^9^ ~ 10^10^	1 (0.71)	0	1 (1.35)	2 (0.85)
10^8^ ~ 10^9^	7 (4.96)	2 (10.00)	1 (1.35)	10 (4.26)
10^7^ ~ 10^8^	4 (2.84)	4 (20.00)	2 (2.70)	10 (4.26)
10^6^ ~ 10^7^	5 (3.55)	2 (10.00)	9 (12.16)	16 (6.81)
10^5^ ~ 10^6^	19 (13.48)	4 (20.00)	8 (10.81)	31 (13.19)
10^4^ ~ 10^5^	36 (25.53)	5 (25.00)	5 (6.76)	46 (19.57)
Positive ~ 10^4^	32 (22.70)	3 (15.00)	19 (25.68)	54 (22.98)
Number of positive lots	104 (73.76)	20 (100.00)	45 (60.81)	169 (71.91)
Number of negative lots	37 (26.24)	0	29 (39.19)	66 (28.09)
Number of lots tested	141	20	74	235

**Fig. 3:**
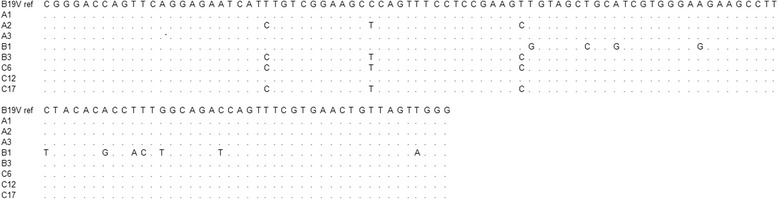
Multiple sequence alignment of the B19V prototype isolate [GenBank: M13178.1] and isolates identified in pools

To confirm the accuracy of the NAT procedure, PCR products of the positive samples were cloned into pMD18-T vector (Takara Bio, Dalian, China) and sequenced using ABI 3730 and accessories. Then the sequences were aligned with the sequence of B19V prototype isolate (GenBank accession no. M13178.1) using DNAStar software package. Sequencing and alignment of amplification products of the positive samples revealed that they exhibited 90.24 %~100 % identity with the prototype (Fig. 3).

In conclusion, the data present demonstrates a relatively high prevalence of B19V in Chinese plasma pools. Therefore, to reduce the risk of B19V transmission, the implementation of B19V NAT assays capable of detecting all B19V genotypes and discard donations with high titer B19V DNA for Chinese blood products manufacturers seems to be necessary.

## Abbreviations

B19V: Human parvovirus B19
qPCR: Quantitative polymerase chain reaction
NAT: Nucleic acid testing
PPTA: Plasma Protein Therapeutics Association
WHO: World Health Organization
FDA: Food and Drug Administration
Ph. Eur.: European Pharmacopoeia
NIBSC: National Institute for Biological Standards and Control
IC: Internal control
